# Ras1 Acts through Duplicated Cdc42 and Rac Proteins to Regulate Morphogenesis and Pathogenesis in the Human Fungal Pathogen *Cryptococcus neoformans*

**DOI:** 10.1371/journal.pgen.1003687

**Published:** 2013-08-08

**Authors:** Elizabeth Ripley Ballou, Lukasz Kozubowski, Connie B. Nichols, J. Andrew Alspaugh

**Affiliations:** 1 Department of Medicine, Duke University School of Medicine, Durham, North Carolina, United States of America; 2 Molecular Genetics and Microbiology, Duke University School of Medicine, Durham, North Carolina, United States of America; University of California San Francisco, United States of America

## Abstract

Proliferation and morphogenesis in eukaryotic cells depend on the concerted activity of Rho-type GTPases, including Ras, Cdc42, and Rac. The sexually dimorphic fungus *Cryptococcus neoformans*, which encodes paralogous, non-essential copies of all three, provides a unique model in which to examine the interactions of these conserved proteins. Previously, we demonstrated that *RAS1* mediates *C. neoformans* virulence by acting as a central regulator of both thermotolerance and mating. We report here that *ras1Δ* mutants accumulate defects in polarized growth, cytokinesis, and cell cycle progression. We demonstrate that the *ras1Δ* defects in thermotolerance and mating can be largely explained by the compromised activity of four downstream Rho-GTPases: the Cdc42 paralogs, Cdc42 and Cdc420; and the Rac paralogs, Rac1 and Rac2. Further, we demonstrate that the separate GTPase classes play distinct Ras-dependent roles in *C. neoformans* morphogenesis and pathogenesis. Cdc42 paralogs primarily control septin localization and cytokinesis, while Rac paralogs play a primary role in polarized cell growth. Together, these duplicate, related signaling proteins provide a robust system to allow microbial proliferation in the presence of host-derived cell stresses.

## Author Summary

Infection with the organism *Cryptococcus neoformans* leads to inflammation of the brain and is lethal if left untreated. Our efforts to understand the ability of this fungus to grow in the human body have lead us to a model for cell growth that is applicable both to other pathogens and to human biology. In earlier work, we examined a protein called Ras1 and showed that *Cryptococcus* uses Ras1 to survive and grow within the human body. In this work, we examine the connection between Ras1 and two other related proteins, Cdc42 and Rac. We have found that these three protein families interact in various combinations to allow the fungal cell to proliferate, to maintain its proper cell shape, and to mate, each of which is an important aspect of the cryptococcal life cycle. These same proteins have been shown by others to be important in human cell growth and malignant transformation. This work provides insight that will both advance our understanding of fungal pathogens and, by providing a simple system in which to test protein function, aid efforts to better understand mammalian cell growth and differentiation, particularly the changes that occur during human cancer progression.

## Introduction

Ras GTPases are central regulators of cell proliferation in all eukaryotes, and perturbations that impair Ras signal transduction have profound effects on cellular morphogenesis and function. In mammalian cells, activating mutations of *RAS* genes result in malignant transformation via multiple anti-apoptotic and proliferative pathways [1]. Analogously, activating Ras mutations in fungi lead to altered morphogenesis and increased substrate invasion [2]–[5]. In each instance, these Ras mutations stimulate proliferation and invasion through the aberrant regulation of multiple downstream effectors. Likewise, proliferation is repressed by the loss of Ras function. For example, *Cryptococcus neoformans ras1Δ* mutants exhibit defects in polarized growth and cell cycle progression, arresting as large, round cells upon exposure to temperature stress [2].

Recent work in various fungal species has demonstrated the potential impact of studying complex Ras signaling paradigms in simple model systems. Ras proteins in both fungi and mammals often act through small Rho-like GTPases (Rac, Rho, or Cdc42) [6]–[9]. Investigators studying the fission yeast *Schizosaccharomyces pombe* have demonstrated that Ras effector specificity is precisely regulated via the subcellular localization of Ras either to the plasma membrane, where it activates a MAP kinase cascade, or to endo-membranes, where it interacts with Cdc42 to support cytoskeletal organization [10], [11]. Similar divisions of Ras function based on subcellular localization have also been demonstrated both in mammalian cells and pathogenic fungi [7], [12].

Despite their utility in identifying conserved Ras interactions, the model yeasts may be limited as predictive models of Ras signaling in other organisms. For example, Ras activation of the cAMP pathway appears to be unique to *Saccharomyces cerevisiae* and its most closely related species [13]–[16]. Moreover, simple yeasts such as *S. pombe* and *S. cerevisiae* lack Rac homologs, instead utilizing single Cdc42 homologs to direct both cell division and differentiation. In contrast, filamentous fungi often have Ras, Cdc42, and Rac homologs, making them well suited for the exploration of more complex signaling interactions [16]–[18].

Previously, we proposed a model of *C. neoformans* Ras signal transduction in which Ras-dependent morphogenesis is mediated by both Cdc42 and Rac proteins [19]. This paradigm has also been studied in other morphologically complex fungi, most notably in *Penicillium marneffei*, where *Pm*RasA is essential for viability [18], [20]. *C. neoformans*, in contrast, demonstrates remarkable plasticity with regard to Rho-GTPase function: neither Ras, Rac, nor Cdc42 paralogs are individually essential, making *C. neoformans* an excellent system in which to study these complex interactions and their contributions to cell proliferation and development [2], [19], [21]–[23].

As an important agent of a human infectious disease, *C. neoformans* also serves as a model for the role of Ras proteins in pathogenesis. Ras proteins control multiple aspects of morphogenesis, development, and cell stress response in *C. neoformans* and other pathogens, and many of these processes are required for virulence in the host [2], [3], [18], [24]–[27]. *C. neoformans* Ras1 activity is required for growth at 37°C and for hyphal development under filamenting conditions [17]. Additionally, Ras1 is required for *C. neoformans* mating processes, including pheromone sensing, cell fusion, and MAP kinase pathway activation [2], [28]. As in *S. pombe*, *C. neoformans* Ras1 directs these distinct functions via differential protein localization: thermotolerance requires Ras1 plasma membrane localization, while endo-membrane-localized Ras1 is sufficient to support mating [12]. Unlike other model fungal systems, the haploid *C. neoformans* genome encodes duplicate copies of *RAS (RAS1/RAS2)*, *CDC42* (*CDC42/CDC420*), and *RAC* (*RAC1/RAC2*) genes. This gene duplication event is conserved among *Cryptococcus* species and varieties, but it does not appear to be present in other basidiomycetes or in more distantly related fungi and is particularly intriguing given the lack of evidence of a whole genome duplication in this organism [19], [23], [29].

Previous work from our lab and others has suggested that there is a regulatory relationship in fungi between Ras and Rac or Cdc42 [16]–[18]. The over-expression of *CDC42*, *CDC420*, *RAC1*, or *RAC2* suppresses *ras1Δ* defects in thermotolerance, consistent with a model in which these GTPases function downstream of Ras1 [17], [22]. However, the terminal phenotype of the *ras1Δ* mutant incubated at elevated temperatures – that of a large, uni-nucleate, unbudded cell – is distinct from those of the individual, putative Ras1 effector mutants [17], [19], [23]. The loss of Cdc42 function results in the accumulation of cytokinesis defects due to loss of the organization of septin proteins: *cdc42Δ cdc420Δ* mutants arrest as multinucleate, un-separated chains of cells [19]. *Cn*Rac1 and Rac2 play a primary role in polarized growth, specifically in hyphal development during mating and in vesicle transport in the yeast phase [22], [23], [30].

Based on this discrepancy, we hypothesized that the terminal *C. neoformans ras1Δ* phenotype is the result of cumulative defects in separable signal transduction pathways. To address this hypothesis, we undertook a more detailed examination of *ras1Δ* defects and report here that *ras1Δ* mutants have defects in cell polarity, cytokinesis, and cell cycle regulation, especially in the presence of cell stress. We demonstrate that the *ras1Δ* defects in thermotolerance can be largely explained by disordered Cdc42 and septin protein function, while hyphal morphogenesis, cell polarity, and cell cycle regulation are primarily mediated by Rac GTPases. Therefore, the separate GTPase classes play distinct primary roles in *C. neoformans* morphogenesis and pathogenesis.

## Results

### Ras1 activates paralogous Rac and Cdc42 proteins in *C. neoformans*

In our search for Ras1 effectors of pathogenesis during *C. neoformans* infection, we identified two Cdc42 proteins (Cdc42/Cdc420) and two Rac proteins (Rac1/Rac2) [19], [23]. Although they are highly related GTPases, Cdc42 and Rac proteins have distinct protein features. Both CnRac proteins have a tryptophan residue at position 59 that defines this class of GTPases [31]. Additionally, the Rac and Cdc42 proteins in *C. neoformans* contain divergent switch I/II regions, which have been shown to be important in interactions with related signaling proteins [32], [33]. Such sequence differences suggest functional differentiation between these related protein classes. In fact, previous work from our group suggests that the CnRac proteins are primarily involved in cell polarization events, while the CnCdc42 proteins are more specialized to direct cytokinesis and septin localization. However, the distinct and overlapping roles of these related GTPases, especially as effectors of Ras protein signaling, have only been indirectly tested [19], [22], [23], [30].

**Figure 1:**
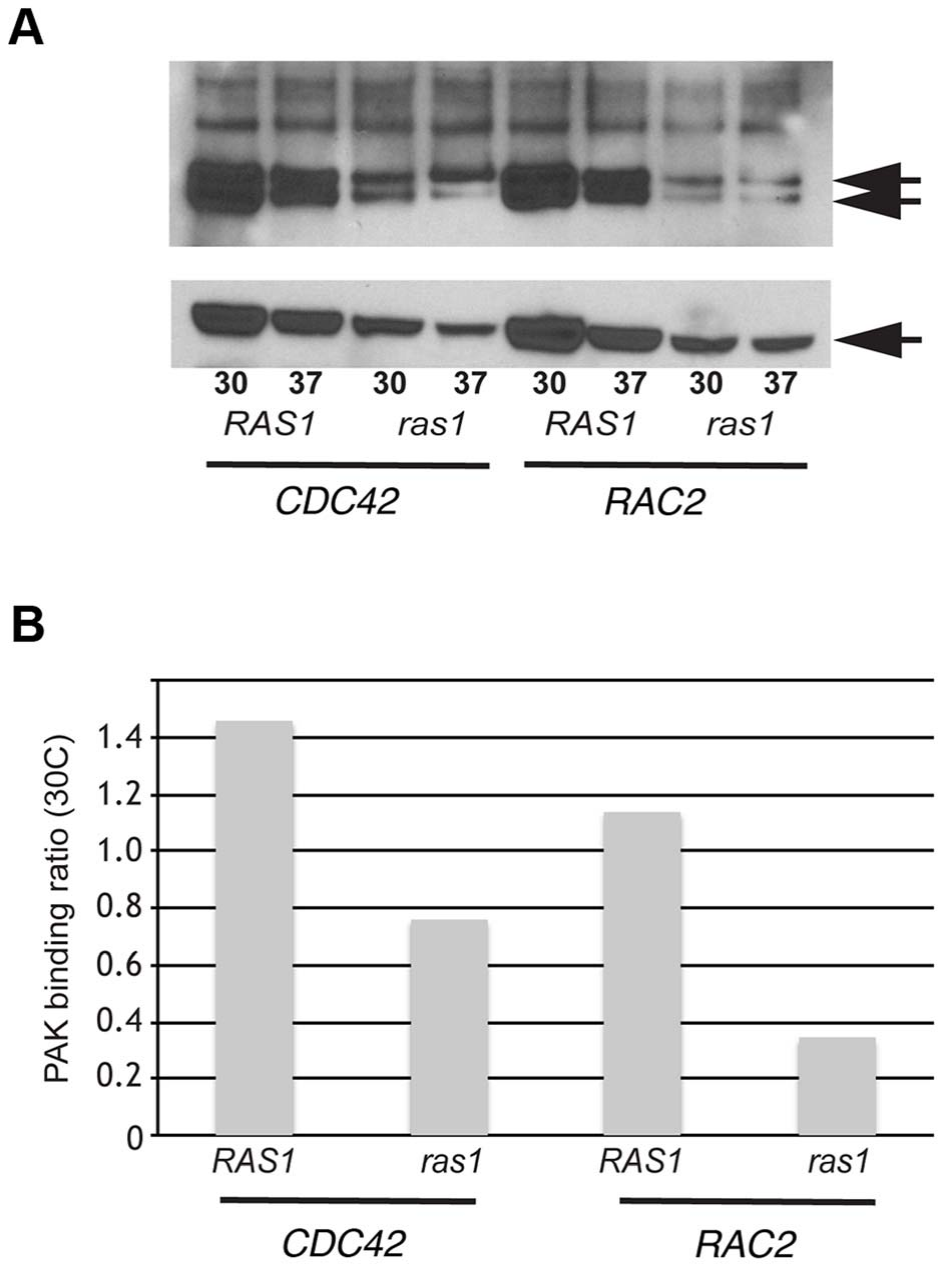
Ras1 is required for Cdc42/Rac-mediated PAK activation. (A) The *ras1Δ* mutant strain and a wild type strain (H99) were transformed with plasmids constitutively expressing *GFP-CDC42* and *GFP-RAC2*. Representative transformants were grown overnight, diluted into fresh media, and incubated at 30°C or 37°C for four hours. Cell lysates were prepared, quantified, and divided between a loading control sample and an experimental sample. Each experimental sample lysate was incubated with GST-PAK-PBD to specifically bind GTP-bound, active Cdc42 or Rac2 and precipitated using glutathione beads. Experimental (top panel) and control (bottom panel) samples were separated by NuPAGE electrophoresis and transferred to PVDF. Immunoblot analysis with anti-GFP antibody revealed bands that migrated at the predicted molecular weight for GFP-Cdc42 and GFP-Rac2 (50 kDa, indicated in each panel). Doublets in the experimental samples represent known changes in Cdc42 mobility following PAK analysis. (B) Signal intensity ratios were determined using ImageJ.

We used a biochemical assay for GTPase effector binding to directly test the model that CnRas1 is required for the activation of Rac and Cdc42 GTPases. Interactions between Rho-GTPases and the CRIB domain of the p21-activated kinase 1 (Pak1) protein are highly conserved. Based on sequence similarity with other known Pak1-binding proteins, both CnRac and CnCdc42 are predicted to efficiently bind mammalian Pak1 when in the GTP-bound, but not the GDP-bound form [34]. In a pull-down assay using a truncated Pak1-Protein Binding Domain (PBD) construct bound to glutathione beads, we detected binding of the Pak1-PBD to both CnRac and Cdc42 proteins (Figure 1A). The intensity of the resulting signal by western blotting was quantified by densitometric analysis using ImageJ and expressed as a ratio to total tagged Rho-GTPase signal in the cell lysates without prior immunoprecipitation (PAK binding ratio, Figure 1B). In other systems, this measurement directly correlates with the level of activated Rac/Cdc42 protein [35]. We then determined the role of Ras1 in modulating the activity of Rac and Cdc42 by detecting PAK binding in the *ras1Δ* mutant. The loss of *RAS1* function dramatically reduced the level of PAK binding by both CnRac2 and CnCdc42 detected in this assay (Figure 1). These data indicate that Ras1 plays a central role in the activation of Rac and Cdc42 GTPases in *C. neoformans*. They are also consistent with prior genetic data suggesting functional interactions among these signaling proteins [17], [19], [22], [23].

### Functional characterization of Ras-Rho-GTPase signal transduction cascades

Previous gene disruption experiments suggested that the Cdc42 and Rac proteins play distinct roles in the growth and development of *C. neoformans*, although there may be some minor redundancy in function. A *cdc42Δ cdc420Δ* double mutant displays severe defects in cytokinesis and septin localization [19]. In contrast, mutation of the Cn*RAC* genes results in defects in cell polarization, both in the yeast and hyphal phases of fungal growth [22], [23]. Given the biochemical confirmation of the role of CnRas1 in Rac and Cdc42 protein activation, we examined the interaction of Ras1 and the Rho-GTPases during the cellular processes required for *C. neoformans* survival in the infected host and the environment.

### Ras1 acts through Cdc42 paralogs to control septin localization

**Figure 2:**
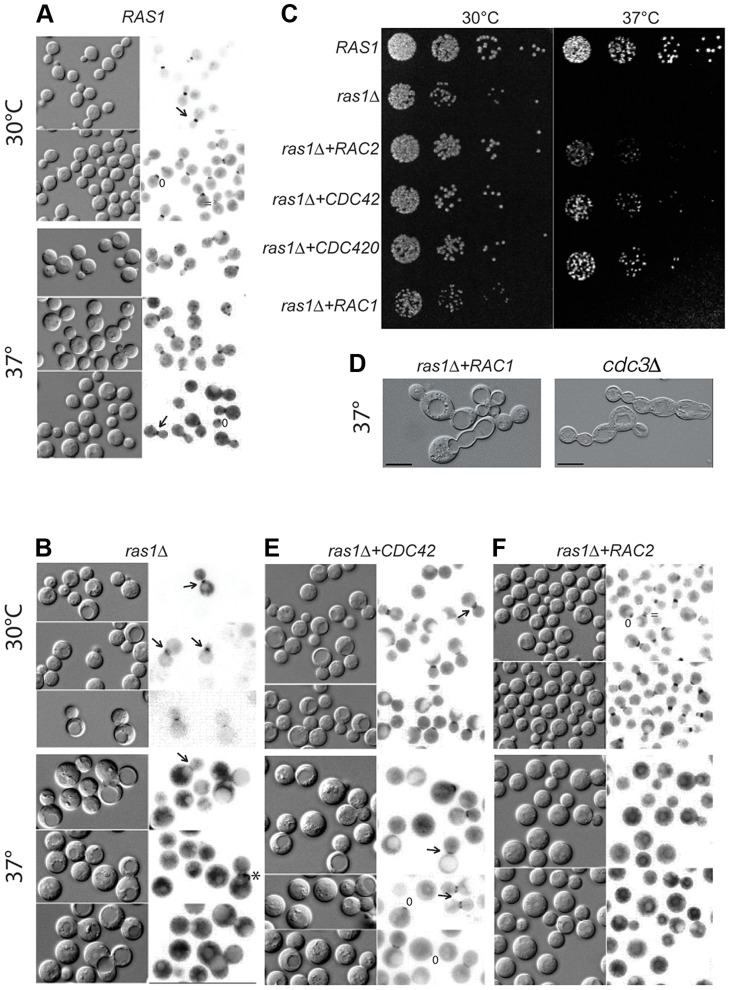
Septin localization in *C. neoformans* cells at 30 and 37°C after 4 hours. Localization of the Cdc11-mCherry fusion construct in various cell backgrounds incubated at the indicated conditions for 4 hours. A) In wild type cells, septin localization undergoes predictable changes in organization from a patch in the early emerging bud (*) to the hourglass arrangement on either side of the neck (←) during bud development, to the double ring structure during cytokinesis ( = ). B) Localization is lost in the majority of *ras1Δ* cells at 37°C, although aberrant localization can be observed in a minority of cells (2.7%). C) Wild type, *ras1Δ*, *ras1Δ+RAC2*, *ras1Δ+CDC42*, *ras1Δ+CDC420*, and *ras1Δ+RAC1* strains were spotted in 5 fold dilutions starting with 10^6^ cells/ml. Plates were incubated for 48 hours at 30°C or 37°C and then photographed. D) The loss of the septin *CDC3* results in the accumulation of cytokinesis defects at both 30 and 37°C, with cells forming chains of un-separated daughters. The over-expression of *RAC1* restores polarized growth and bud development to the *ras1Δ* mutant, but is insufficient to restore cytokinesis. Like *cdc3Δ* cells, *ras1Δ+RAC1* cells accumulate defects in cytokinesis, with daughter cells failing to separate. E) The over-expression of *CDC42* paralogs restores Cdc11-mCherry localization at 37°C. F) The over-expression of *RAC2* fails to restore Cdc11-mCherry localization at 37°C.

In wild type *C. neoformans* cells, four septin proteins (Cdc3, Cdc10, Cdc11, and Cdc12) co-localize to the initial site of bud growth and undergo dynamic and highly conserved changes in organization as bud development transitions from apical to isotropic growth to cytokinesis, similar to those changes described in *S. cerevisiae* [36], [37]. These transitions can be observed as changes in localization from a patch at the site of bud emergence, to an hourglass during bud growth, and finally to two separate rings on either side of the bud neck during cytokinesis. In wild type strains, this process is indistinguishable at 30°C and 37°C (Figure 2A) [19]. Mutation of the individual septin proteins and the subsequent loss of septin complex organization results in defects in cytokinesis at 37°C [37].

We previously demonstrated that Cdc42 and Cdc420 are required for septin protein localization and efficient cytokinesis under host-specific conditions [19]. We therefore hypothesized that the loss of Ras1-mediated activation of Cdc42 proteins might negatively impact septin organization. To test this model, we assessed the role of Ras1 in Cdc42-mediated septin assembly by observing the localization of a Cdc11-mCherry fusion protein in the *ras1Δ* mutant. When the *ras1Δ* mutant strain was incubated at 30°C, the Cdc11-mCherry fusion protein localized appropriately to the bud neck and was observed undergoing the various structural changes associated with normal bud development (Figure 2B). However, when *ras1Δ* mutant cells were shifted to 37°C for 4 hours, Cdc11-mCherry localization at the bud neck was lost in a majority of cells (97.3%, n = 300) (Figure 2B). Instead, Cdc11-mCherry signal was diffusely localized throughout the cytoplasm and on endo-membranes. Rarely, *ras1Δ* cells exhibited Cdc11-mCherry localization at the bud neck (2.7%); however, in the majority of those cells, localization was inappropriate for the budding state, consistent with a defect in cytokinesis. For example, in Figure 2B the cell indicated (*) demonstrates septins that have failed to transition to the double ring structure prior to the emergence of the new bud. Similar defects in septin localization and cytokinesis are observed in *cdc42Δ* and *cdc420Δ* mutants [19].

To further test which of the Ras1 downstream pathways contributes to Ras-mediated septin function and cytokinesis, we also examined the effect of Cdc42 and Rac protein over-expression on septin localization in the *ras1* mutant background. *CDC42* and *RAC* paralogs were placed under the control of the *HIS3* promoter and transformed into the *ras1Δ* mutant background. Over-expression in the *ras1Δ* background was confirmed by RT-PCR compared to an internal control (*GPD1*) and is reported here compared to relative expression levels from endogenous promoters in the *ras1Δ* background: 7.3-fold over-expression for *CDC42*; 3.7-fold over-expression for *CDC420*; 15.5-fold over-expression for *RAC2*. As previously reported, overexpression of these genes restores thermotolerance to the *ras1Δ* mutant (Figure 2C) [19], [22], [23].

The over-expression of *CDC42* restored septin localization at 37°C in *ras1Δ* mutant cells (Figure 2E), as did the over expression of *CDC420* (data not shown). In contrast, *RAC* paralog over-expression failed to restore cytokinesis or septin localization. Although the over-expression of *RAC2* was sufficient to restore thermotolerance at 37°C, it was not sufficient to restore the localization of the Cdc11-mCherry fusion protein at 37°C in the *ras1Δ* background (Figure 2F). Instead, Cdc11-mCherry remained localized to intracellular structures in the *ras1Δ+RAC2* background at 37°C, similar to its localization in the *ras1Δ* background. Western blotting of the mCherry-Cdc11 construct in the various strain backgrounds at 30 and 37°C revealed no evidence of protein degradation upon temperature shift (data not shown).

### Ras1 acts through Cdc42 paralogs to control bud morphogenesis

Data from *C. neoformans* septin mutants and the *cdc42Δ cdc420Δ* mutant suggests that defects in septin organization such as those described above should impair cytokinesis at high temperature and result in cells with elongated, abnormal buds and/or multi-budded cells [19], [37]. However, prolonged incubation of the *ras1Δ* mutant at 37°C leads to uniformly large, unbudded cells [2]. This, coupled with the biochemical data revealing a role for Ras1 in the activation of both Rac and Cdc42, suggested that the *ras1Δ* proliferation defect might be the result of defects in multiple branches of signal transduction: i.e., both Cdc42 and Rac activity are impaired in the *ras1Δ* mutant, resulting in pleotropic defects.

**Figure 3:**
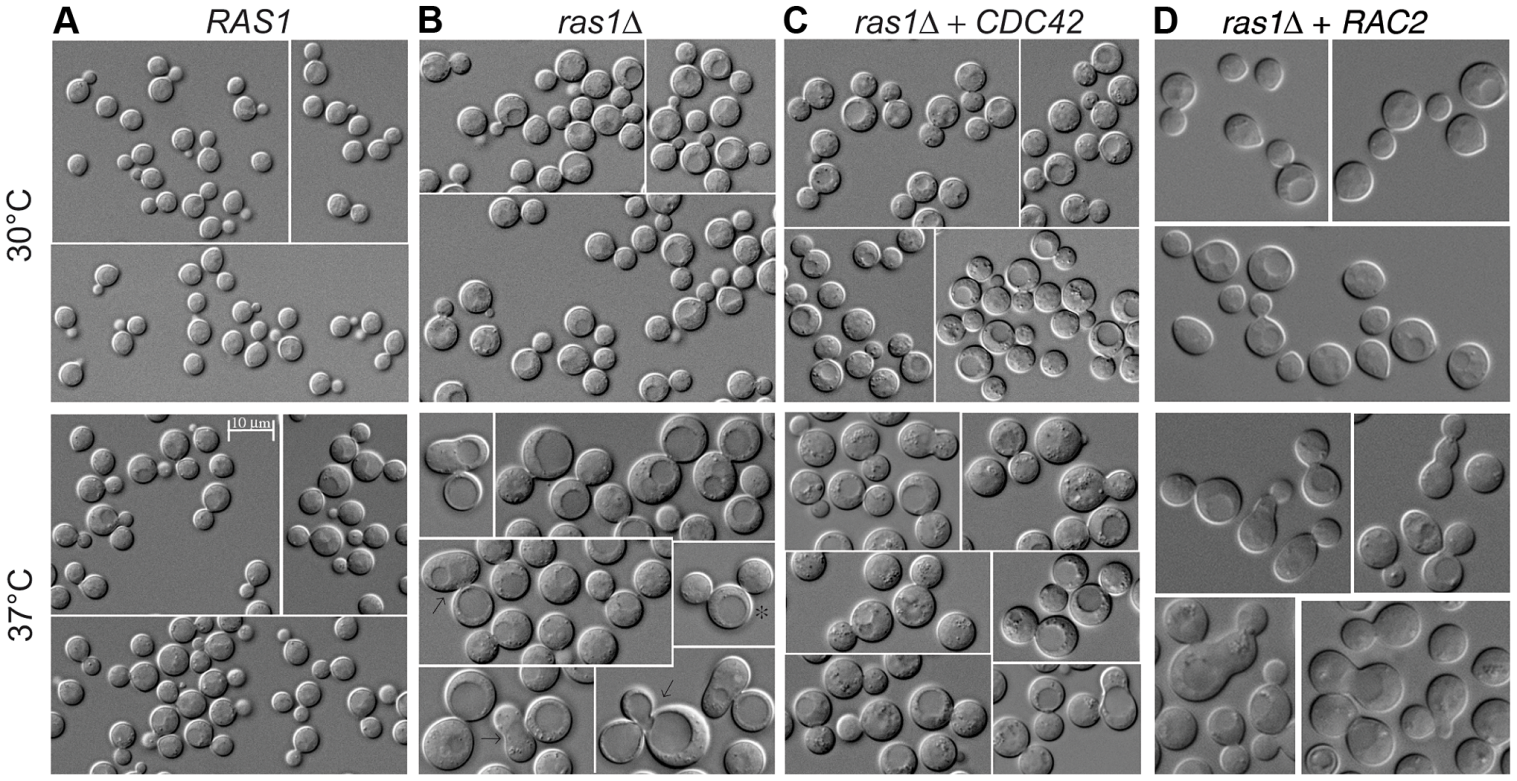
Yeast phase morphology of *C. neoformans* cells at 30 and 37°C after 4 hours. Wild type (A), *ras1Δ* (B), *ras1Δ+CDC42* (C), and *ras1Δ+RAC2* (D) cells were incubated at the indicated conditions for 4 hours. A) Wild type cells proliferate by budding at both 30 and 37°C; however, at 37°C wild type cells increase in size overall compared to 30°C. B) Mutant *ras1Δ* cells are larger than wild type cells at both 30 and 37°C. At 37°C, a population of *ras1Δ* budding cells (57.5% vs. 66.2% in wild type) was observed, 42.9% of which were abnormal in morphology (vs. 2.8% in wild type cells). Abnormal morphology included mother cells with multiple buds (*) or abnormally large buds (8) (>75% as large as mother cells), as well as buds with wide necks or abnormal morphology (←). C) The over-expression of *CDC42* restores wild type morphology to the majority of *ras1Δ* buds at 37°C (72.5% budded, 11.2% abnormal), but does not restore cell size. D) The over-expression of *RAC2* restores cell size to *ras1Δ* cells at 30°C and 37°C, but does not support cell separation. Scale bar = 10 µm for all panels.

We tested this by quantifying the effect of short-term vs. long-term exposure to temperature stress on *ras1Δ* cell morphogenesis. We microscopically examined *ras1Δ* cells following incubation at 37°C for 4 and 24 hours. As previously reported, after 24 hours at 37°C, the majority of *ras1Δ* cells arrested as large unbudded cells, while wild type cells continued to proliferate normally (*ras1Δ*: 91.8% unbudded, 8.2% budded; wild type: 37.5% unbudded, 62% budded, n>100) (Table 1). In contrast to the uniformly large and unbudded appearance of *ras1Δ* cells after 24 hours of incubation at 37°C, microscopic examination of *ras1Δ* mutant cells after 4 hours at 37°C revealed the presence of a population of budding cells (*ras1Δ*: 42.5% unbudded, 57.5% budded; wild type cells: 33.8% unbudded, 66.2% budded; n>200) (Table 1). Of these budding *ras1Δ* cells, 42.9% exhibited clear defects in morphogenesis, compared to 2.8% morphologically aberrant buds in wild type cultures after 4 hours at 37°C. These defects included mother cells with multiple buds or abnormally large buds (>75% as large as mother cells), as well as buds with wide necks or abnormal morphology. Representative images of these abnormally budded cells are presented in Figure 3B. These data are consistent with a defect in septin organization following a temporary stress in the absence of *RAS1*.

**Table 1:** Analysis of bud morphology.

		% Unbudded	% Budded	
37°C, 4 hrs	*RAS1*	**33.8**	**66.2**	n_b_ = 141 budded
	n_t_ = 213		97.2	*% normal bud*
			2. 8	*% abnormal bud*
	*ras1Δ*	**42.5**	**57.5**	n_b_ = 177 budded
	n_t_ = 308		56.1	*% normal bud*
			42.9	*% abnormal bud*
	*ras1Δ+*	**27.5**	**72.5**	n_b_ = 179 budded
	*CDC42*		88.8	*% normal bud*
	n_t_ = 247		11.2	*% abnormal bud*
	*ras1Δ+*	**51.1**	**48.9**	n_b_ = 161 budded
	*CDC420*		86.3	*% normal bud*
	n_t_ = 329		13.7	*% abnormal bud*
	*ras1Δ+*	**79.7**	**20.3**	n_b_ = 144 budded
	*RAC2*		66.6	*% normal bud*
	n_t_ = 709		33.3	*% abnormal bud*
37°C, 24 hrs	*RAS1*	37.5	62.5	n = 152
	*ras1Δ*	91.8	8.2	n = 146

Log-phase cultures of the indicated cell types were incubated at 37°C for 24 hours or 4 hours in liquid medium, 150 RPM. DIC images of the cultures were analyzed for mother and daughter cell morphology. For 4 hour cultures, a sufficient number of cells (n_t_) was examined to identify comparable numbers of budding cells (n_b_). Abnormal buds included those that were non-round or had wide bud necks, and mother cells with multiple buds or buds that were abnormally large (>75% of the size of the mother cell).

Consistent with its ability to restore septin protein localization to the *ras1Δ* mutant, Cdc42 over-expression also suppressed *ras1Δ* mutant defects in budding after 4 hours of growth at 37°C. Unlike *ras1Δ* cells, in which approximately 50% of buds appeared abnormal after 4 hours incubation at 37°C, the *ras1Δ+CDC42* (Figure 3C) and *ras1Δ+CDC420* (data not shown) mother cells formed buds that appeared normal in morphology at this elevated temperature (*ras1Δ+CDC42*: 72.5% budded, of which 11.2% were abnormal; *ras1Δ+CDC420*: 48.9% budded, of which 13.7% were abnormal; n>200. Table 1).

In contrast, the over-expression of *RAC* paralogs did not restore bud morphology. Although the over-expression of *RAC2* in the *ras1Δ* background partially restored high temperature growth and mitigated the large cell size of the *ras1Δ* mutant, microscopic examination of the cells confirmed that abnormal bud morphogenesis persisted (20.3% budded, of which 33.3% were abnormal; n>700) (Table 1) (Figure 3D) (Note that sufficient cells were counted to achieve similar numbers of budded cells across strains). These data suggest that *RAC* paralogs play no native role in the organization of septin proteins. In fact, neither *rac1Δ* nor *rac2Δ* deletion strains exhibit defects in septin localization at 30°C or 37°C (data not shown), nor do they exhibit defects in cytokinesis [23].

To more fully explore the effects of *RAC* gene overexpression in the *ras1* strain, we independently generated a strain with only 3.7-fold over-expression of *RAC1* in the *ras1Δ* background. This lower level of *RAC1* expression was able to restore hyphal morphogenesis to the *ras1Δ* mutant (discussed below) but was insufficient to restore thermotolerance (Figure 2C). *RAC1* over-expression at higher levels has been previously demonstrated to restore thermotolerance to the *ras1Δ* mutant [22]. Interestingly, this *ras1Δ+RAC1* strain with relatively low *RAC1* over-expression resulted in cells that no longer arrested as large unbudded cells like the *ras1Δ* background strain. Instead, they demonstrated profound defects in cytokinesis at 37°C, closely phenocopying *cdc3Δ* septin deficient cells (Figure 2D) [37]. Therefore, low-level *RAC* gene overexpression is able to restore normal cell size to the *ras1Δ* strain, but it is unable to complement this strain's defects in cytokinesis or septin localization.

These data are consistent with a role for Ras1 in the regulation of Cdc42-mediated septin organization and suggest a model in which the loss of Ras-mediated activation of Cdc42, but not Rac, impairs the organization of septins and results in the accumulation of defects in bud morphology after short-term exposure to 37°C.

### Ras1 is required for cell cycle control and acts through Rac

**Figure 4:**
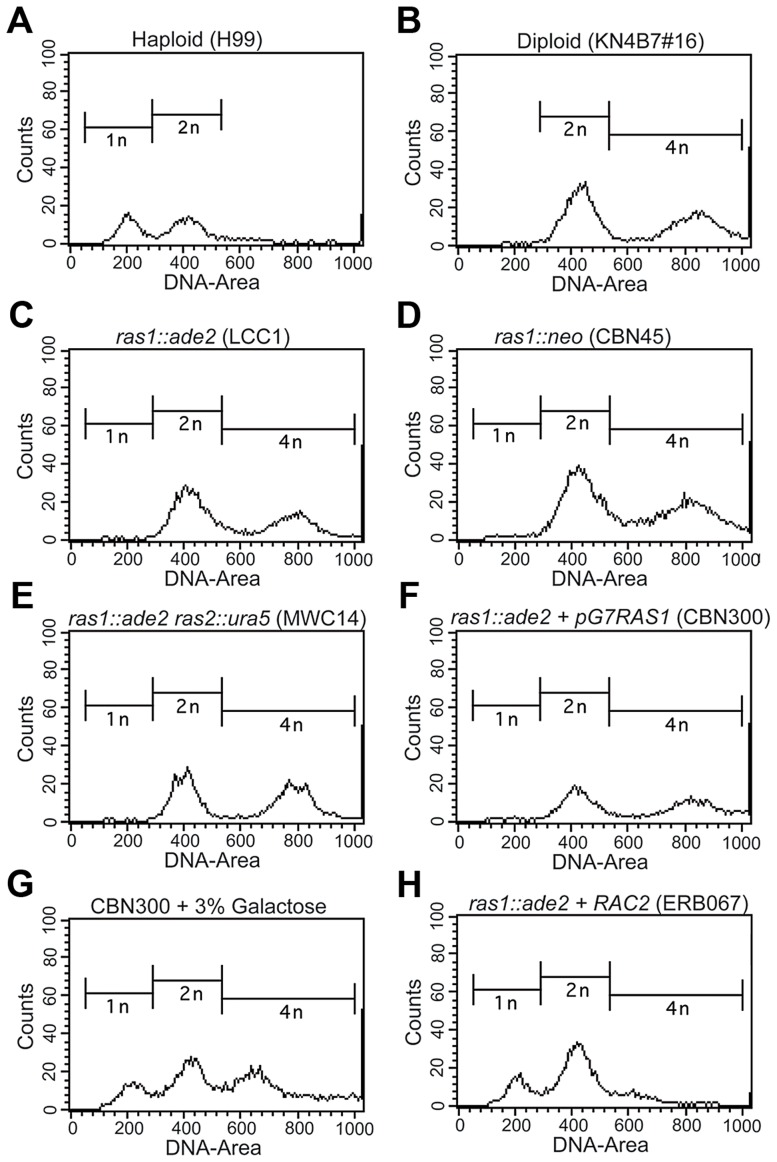
The loss of *RAS1* impacts cell ploidy. Yeast phase cells from log-phase cultures were grown for four hours at 30°C, and then fixed and prepared for the assessment of DNA content by FACS analysis. Control haploid (A) and diploid (B) strains exhibited peaks at the expected ratios for haploid and diploid content. Independently generated *ras1Δ* strains, as well as the *ras1Δ ras2Δ* strain, (C–F) were found to more closely resemble the diploid strain than the haploid strain. G) When a *ras1Δ+GALRAS1* strain was incubated for 24 hours under galactose inducing conditions, the emergence of a population of haploid-like cells was observed. H) The over-expression of *RAC2* in the *ras1Δ* background restored haploid-like ploidy to the *ras1Δ* mutant strain.

Because bud emergence and septin organization are closely linked with cell cycle control in the model budding yeast, we examined *ras1Δ* mutant cells for defects in cell cycle control [38]. Two independently generated *ras1Δ* mutants were examined for nuclear content by FACS analysis (Figure 4). At 30°C, both haploid (H99) and diploid (KN4B7#16) populations exhibited biphasic DNA content distributions corresponding to G1 and G2 phase cells (Figure 4A, B) [39]. H99 peaks corresponded to 1N or 2N content, while KN4B7#16 peaks corresponded to 2N and 4N content. Interestingly, two independently generated *ras1Δ* strains, as well as a *ras1Δ ras2Δ* strain, when examined during log phase growth at 30°C, were found to most closely resemble diploid (KN4B7#16) cells, with biphasic distributions centered around 2N and 4N (Figure 4C, D, E, F).

In *C. neoformans*, the emergence of a bud precedes nuclear division, which occurs across the bud neck, and cytokinesis occurs rapidly after nuclear division ([40] for review). Therefore, the presence of unbudded, multinucleate cells would suggest altered cell cycle control. In order to account for the possibility that multinucleate cells were responsible for the apparent shift in nuclear content, more than 100 cells from each condition were examined microscopically. In a population of log-phase wild type haploid control cells, 74% were unbudded or budded and uni-nucleate, 26% were budded and bi-nucleate, 0% were unbudded and bi-nucleate, and 0% were budded and multi-nucleate. Diploid control cells showed a similar distribution (77%, 23%, 0%, 0%) at 30°C. Changes in *ras1Δ* ploidy at permissive temperatures were not the result of accumulating multinucleate cells, as *ras1Δ* strains exhibited similar budding indices and similar nuclear distribution to both *RAS1* competent strains (73%, 27%, 0%, 0%, n>100).

We examined the role of Ras1 activity in mediating ploidy by observing the effect of *RAS1* induction on the *ras1Δ* mutant population. We constructed a *ras1Δ* strain in which the *RAS1* gene was placed under the control of a galactose inducible promoter and observed that the altered ploidy of the *ras1Δ* mutant strain is partially reversible (Figure 4F, G). Under non-inducing conditions, the *ras1Δ+pGAL-RAS1* strain behaved similarly to the *ras1Δ* strains (Figure 4F). After 24 hours under inducing conditions and restoration of *RAS1* expression, a peak emerged corresponding to 1N nuclear content (Figure 4G).

These data suggest that the loss of *RAS1* function impacts ploidy relative to wild type haploid cells. Given the ability of the Rho-GTPases to restore yeast phase morphogenesis to the *ras1Δ* mutant, we examined their role in *RAS1*-mediated cell cycle control. Neither *CDC42* paralog deficient nor *RAC* paralog deficient cells exhibited altered ploidy relative to wild type haploid cells (data not shown). Interestingly, while strains over-expressing the *CDC42* paralogs failed to restore haploid nuclear content (data not shown), the over-expression of *RAC2* in the *ras1Δ* background was sufficient to restore ploidy consistent with that of the haploid strain (Figure 4H). As previously described, these *ras1Δ+RAC2* cells are reduced in size compared to the *ras1Δ* strain, and, although they exhibit morphological defects and mis-localized septin proteins consistent with a failure to restore Cdc42-mediated septin organization, they are able to undergo cytokinesis (Figure 3D). The accumulating cytokinesis defects of the *ras1Δ+RAC1* strain, which primarily exists as chains of un-separated cells, made it unsuitable for FACS analysis (Figure 2). Together, these data suggest that *ras1Δ* defects in cell cycle control are the result of defects in Rac-mediated processes and are independent of Ras1-Cdc42 mediated septin organization.

### Ras1 acts through Rac but not Cdc42 paralogs to regulate polarized growth

Strains of the budding yeast *S. cerevisiae* that are compromised in the establishment of cell polarity fail to initiate budding and continue to grow spherically, leading to cell size enlargement [41]. In *C. neoformans*, the phenotype of the *ras1Δ* mutant at 37°C (large unbudded cells) is consistent with a defect in the establishment of cell polarity. Indeed, *ras1Δ* cells at 37°C have a documented defect in actin polarization, especially in the re-establishment of cell polarity after cell stress [17]. We previously demonstrated that mutation of either the *RAC1* or *RAC2* genes results in increased yeast cell size, consistent with the enlarged cell phenotypes of polarity mutants in *S. cerevisiae* [23]. Moreover, Rac paralogs exhibit Ras-dependent localization during yeast-phase growth at 37°C [23]. The double *rac1Δ rac2Δ* mutant appears to be lethal, impairing further investigation of their role in polarized growth during yeast-phase development. However the Rac paralogs are individually required for polarized growth during hyphal development. We therefore used the highly polarized hyphal state of cryptococcal mating to further explore the Ras1-Rac signaling module.

**Figure 5:**
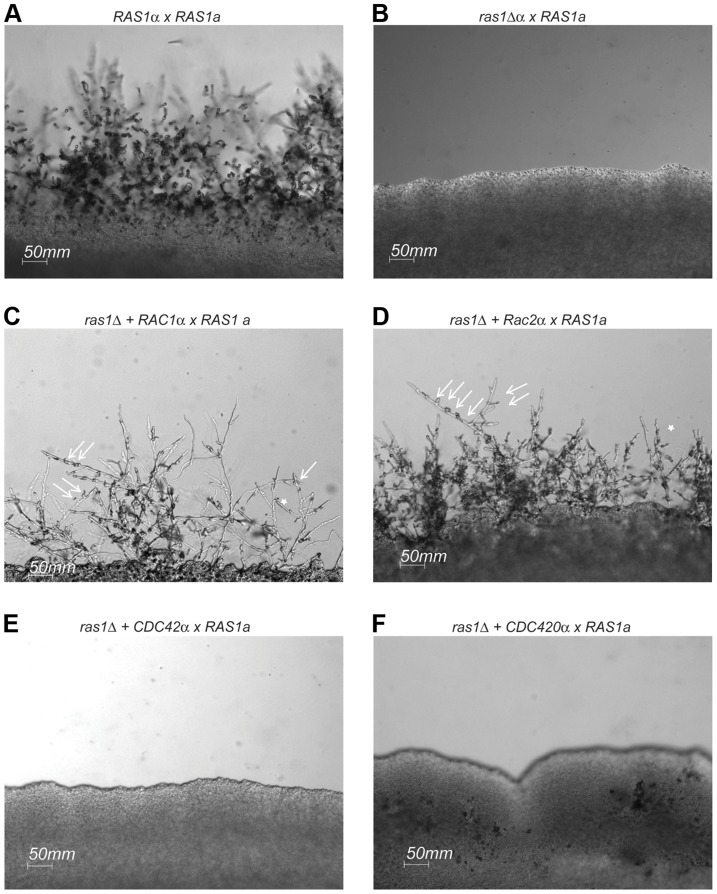
Mating in *ras1Δ+RhoGTPase* strains. Wild type (A), *ras1Δ* (B), *ras1Δ+RAC1* (C), *ras1Δ+RAC2* (D), *ras1Δ+CDC42* (E), and *ras1Δ+CDC420* (F) mating type α cells were co-incubated with wild type mating type **a** cells on MS mating medium for 4 days. Wild type mating reactions results in the formation of hyphal filaments that extend into the medium, while *ras1Δ* cells are sterile. The over-expression of *RAC* paralogs restores filamentation. Arrows indicate abnormal clamp connections (←), and stars (*) indicate fused clamp connections. The over-expression of *CDC42* paralogs fails to restore filamentation to the *ras1Δ* strain. Scale bar = 50 µm.

The *C. neoformans ras1Δ* mutant strain is sterile and is unable to transition to filamentous growth during either a wild type unilateral or a bilateral *ras1Δ* mutant cross (Figure 5). This *ras1Δ* mating defect is multifactorial, involving failures in pheromone gene induction as well as impaired development of the initial polarized germ tubes required for cell fusion [28]. The over-expression of *RAC1* was previously demonstrated to restore hyphal development to the *ras1Δ* mutant in the *C. neoformans* var. *neoformans* Serotype D strain background [22]. We recapitulated this restoration of cell polarity by Rac protein overexpression in the highly virulent Serotype A strain background. While *ras1Δ* cells fail to form hyphae on mating medium in the presence of a wild type mating partner, the over-expression of either *RAC1* or *RAC2* in the *ras1Δ* mutant background is sufficient to restore mating and hyphal development (Figure 5). In contrast, the over-expression of *CDC42* paralogs failed to restore hyphal development to the *ras1Δ* mutant (Figure 5), even though these strains display complemented thermotolerance. This suggests that the effects of Ras proteins on polarized growth during hyphal development are primarily mediated by the Rac paralogs. This observation is further supported by examination of a gain-of-function allele of Ras1, Ras1^Q67L^, which imparts filamentation on filament agar to the H99 background [2]. This gain-of-function was partially impaired by loss of Rac paralogs. Conversely, the over-expression of Rac and Cdc42 paralogs was insufficient to stimulate filamentation, suggesting that GTP-bound Ras1 may play a regulatory role in this process (Figure S1).

### Rac proteins fail to support the differentiation of septin-dependent mating structures during *ras1Δ* mating

**Figure 6:**
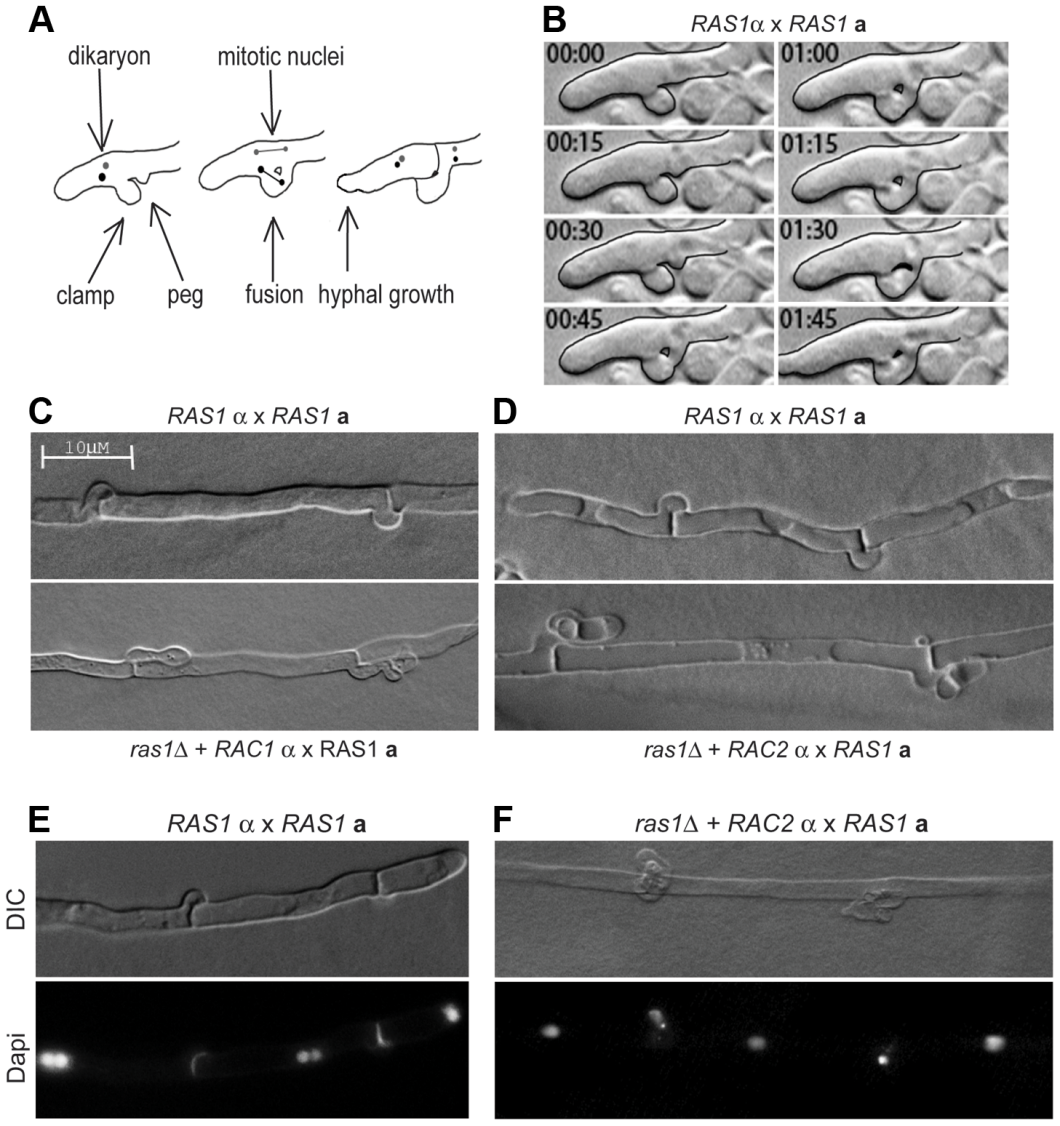
Clamp cell morphogenesis during *C. neoformans* hyphal development. (A) Schematic of clamp cell fusion and nuclear dynamics. (B) Clamp cell morphogenesis depends on the fusion of clamp and peg cells across hyphal compartments. (C, D) The over-expression of *RAC1* (C) or *RAC2* (D) is insufficient to restore clamp cell morphogenesis in the *ras1Δ* background compared to wild type clamp cells (top panels). (E) During *RAS1α*×*RAS1* ***a*** mating, hypha with fused clamp cells are dikaryotic. (F) In mating filaments produced by the *ras1Δ+RAC2α×RAS1* ***a*** cross, nuclei were observed trapped in the unfused clamp cell.

Given the failure of *RAC* paralogs to restore septin organization during yeast phase growth in the *ras1Δ* mutant background, we asked whether the over-expression of *RAC* paralogs was sufficient to restore septin-dependent clamp cell morphogenesis in the absence of *RAS1* [37]. During hyphal development, growth is first directed in a polarized fashion away from the site of initial **a**/α fusion. Second, a specialized cell known as the clamp cell emerges from the distal side of the site of septation and protrudes back towards the proximal compartment (Figure 6). A second protrusion, the peg cell, emerges from the proximal compartment and grows towards the clamp cell. Fusion of the clamp and peg cells results in a clamp connection. This fusion is dependent on the presence of septin proteins, which localize to the site of clamp/peg fusion (Figure S2, [37]).

Microscopic examination of the mating filaments involving *ras1Δ+RAC* paralog strains revealed that the majority of clamp cells had aberrant morphology and had failed to fuse (unfused clamp cell rate for *RAS1α×RAS1* ***a***: 33%; *ras1Δ+RAC1α×RAS1* ***a***: 52%; *ras1Δ+RAC2α×RAS1* ***a***: 66% n>150) (Figure 6C, D). Moreover, the hyphae with abnormal clamp connections terminated in basidia with aberrant spore chains. These morphological defects in mating structures for crosses involving *ras1Δ+RAC1 or ras1Δ+RAC2* strains are similar to those previously observed in crosses involving *cdc42Δ* mutants and *cdc12Δ* septin mutants (unilateral *cdc42Δα*×*CDC42* ***a*** crosses and bilateral *cdc12Δα×cdc12Δ* ***a*** crosses) [19], [37].

Fusion of the clamp and peg cells is essential for the proper division and segregation of dikaryotic nuclei along the elongating hypha [42], [43]. During the initial phase of polarized growth, both nuclei are transported to the distal end of the hypha (Figure 6A schematic). One nucleus undergoes mitosis within the hypha, while the other is transported into the clamp cell. Fusion between the clamp and peg cells provides the nucleus access to the proximal compartment. Mitosis then occurs across the clamp connection, with one nucleus being sent to the distal compartment and one to the proximal compartment. As a result, wild type hyphae with fused clamp connections are dikaryotic (Figure 6E). We examined the *ras1Δ+RAC* paralog strains for defects in nuclear movement consistent with failed clamp cell fusion. For hyphae in which clamp cells had failed to fuse, we observed a single nucleus in the hypha and a second nucleus that had become trapped in the clamp cell projection (Figure 6F). Similar defects in nuclear transport have been observed in both *cdc42Δα*×*CDC42* ***a*** and bilateral *cdc12Δα*×*cdc12Δ* ***a*** crosses [19], [37]. These morphological and nuclear migration defects are consistent with Rac-mediated restoration of hyphal cell polarity but not septin function in the *ras1Δ* background.

One alternative explanation for the un-fused clamp connections we observed in the *ras1Δ+RACα×RAS1* ***a*** crosses is the induction of monokaryotic haploid fruiting. The dikaryotic progression that results from α/**a** fusion is distinct from haploid fruiting, a process characterized by monokaryotic hyphae and unfused clamp connections [44]. To ensure that the hyphal structures observed in the *ras1Δ+RAC* paralog mating reactions were indeed the result of **a**/α fusion, as opposed to haploid fruiting, spores from these crosses were analyzed for mating type and the segregation of genetic markers. Among the rare spores that could be isolated from these crosses, we observed evidence of recombination of genetics markers among spores from both mating types (data not shown). Therefore, despite the presence of frequently un-fused clamp connections, these mating structures were the result of **a**/α mating.

We cannot completely exclude the possibility that the failures we observed in clamp cell fusion were the result of dysregulated polarized growth of the clamp cell due to either *RAC* gene over-expression or due to *RAS1* haploinsufficiency, rather than loss of Cdc42-mediated septin organization. However, when we examined the effect of *RAC1* over-expression on hyphal morphology in the context of intact, wild type levels of Ras1 function, we observed clamp cell fusion defects at rates consistent with wild type clamp fusion, as well as the production of wild type basidia and spore chains, suggesting that the over-expression of *RAC1* alone does not impair morphogenesis during mating (data not shown).

These data suggest that the over-expression of Rac paralogs is sufficient to restore polarized growth but is insufficient to restore Cdc42-mediated septin organization in the *ras1Δ* mutant during both hyphal and yeast phase growth. Conversely, the over-expression of Cdc42 paralogs is sufficient to restore septin- mediated mating events such as clamp cell fusion, but it is insufficient to restore polarized growth required for the development of true hyphae.

## Discussion

Ras1 and the Rho-GTPases control multiple aspects of *C. neoformans* morphogenesis, including polarized growth during both yeast and hyphal growth phases, septin-dependent thermotolerance, and subcellular ROS polarization [17], [22], [23]. The loss of *RAS1* impairs mating, impacts mother and bud size, alters bud morphology, and destabilizes septin proteins. The data presented here suggest that the multifactorial defects in *ras1Δ* morphogenesis reflect the loss of function of multiple downstream effectors, including the Rho-like GTPases Cdc42/Cdc420 and Rac1/Rac2.

Previously, we demonstrated that the over-expression of paralogous Rac and Cdc42 GTPases restores thermotolerance to the *ras1Δ* mutant [17], [22]. Additionally, yeast-two-hybrid experiments indicated an interaction between the activated, GTP-bound form of CnRas1 and the Guanine nucleotide Exchange Factor (GEF) Cdc24 [17]. The GTP-dependent interaction of Ras1 with Cdc24 indirectly supports a role for Ras1 in the localization or regulation of predicted targets of Cdc24 such as the Cdc42 or Rac GTPases [45]. Here, we have provided direct confirmation of Ras1-dependent activation of these proteins in *C. neoformans*. These data are consistent with genetic data in which *C. neoformans cdc24Δ* mutants phenocopy *ras1Δ* mutants in both cell morphology and thermo-sensitivity [17]. In contrast, *C. neoformans cdc42Δ cdc420Δ* mutants do not phenocopy either the *cdc24Δ* or the *ras1Δ* mutant morphology. Although all three mutants are thermo-sensitive, the loss of either *RAS1* or *CDC24* results in large, apolar cells at 37°C, while the loss of *CDC42* paralogs results in cells with defects in cytokinesis. [17], [19]. In contrast, the loss of individual *RAC* paralogs impacted cell size, similar to the loss of *RAS1* or *CDC24*; however, neither *rac1Δ* nor *rac2Δ* mutants are thermo-sensitive [22], [23]. This inconsistency in the individual mutant phenotypes of the proposed Ras1-Cdc24-Cdc42 cascade suggests that Ras1 might, via Cdc24, regulate the activity of multiple GTPases.

Data from other eukaryotic model systems support the emerging paradigm that Ras proteins act through both Cdc42 and Rac to influence cell proliferation and morphogenesis. Rac- and Cdc42-dependent functions for the Ras oncogene were first demonstrated in mammalian cancer cell lines in the late 1990s [8], [9]. Oncogenic Ras mutations are inhibited by the loss of Rac or Cdc42 function, likely via independent pathways [8], [9]. Likewise, previous work from our lab and others has suggested that there is a regulatory relationship in fungi between Ras and the Rho-GTPases Cdc42 or Rac [16]–[18]. However, the identification of a fungal model in which to study the potential for cross talk and specificity between Ras/Rac- and Ras/Cdc42-mediated signal transduction has remained a challenge. For example, while *S. cerevisiae* and *S. pombe* have provided insight into Ras/Cdc42 signal transduction, both organisms lack Rac homologs, limiting their utility for examining Cdc42/Rac cross talk [10], [14], [46]–[49].

Rac1 and Cdc42 homologs have been identified in the related ascomycete *Candida albicans*, where *Ca*Rac1 has been shown to mediate invasive filamentous growth via a MAPK cascade, similar to the activity of human Rac1 [20], [27], [50]. However the regulatory relationship between *Ca*Ras1 and *Ca*Rac1 remains unclear, since *Ca*Rac1 does not play a role in serum-induced filamentation, a major signal for *Ca*Ras1-mediated filamentous growth [20]. Additionally, while both *Ca*Ras1 and *Ca*Cdc42 mediate the yeast-to-hyphal transition in response to serum inducing conditions, the integration of *Ca*Cdc42 and *Ca*Ras1 activity has not yet been definitively demonstrated [20], [51]. Studies of *Ca*Cdc42 point mutants have demonstrated a role for *Ca*Cdc42 activity in reinforcing the transcription of target genes that have also been shown to be induced via *Ca*Ras1-dependent cAMP/PKA signaling [3], [52], [53]. However, despite shared transcriptional targets, *Ca*Ras1 and *Ca*Cdc42 may not directly interact, as we have proposed in *C. neoformans*. For example, while dominant active *Ca*Ras1 induces filamentation, even in the absence of external stimuli, dominant active *Ca*Cdc42 induces accumulated cell morphology defects specifically during yeast-phase growth and cytokinesis [54], [55]. This indicates that, although *Ca*Ras1 and *Ca*Cdc42 may share downstream targets important for hyphal development, they do not necessarily act in a linear pathway.

In the human fungal pathogen *Penicillium marneffei*, which causes disseminated disease in AIDS patients, RasA has been shown to act through cflA (Cdc42) to control yeast cell polarization and conidial germination [18]. However, no clfB (Rac)-dependent function for RasA has been demonstrated. Additionally, *P. marneffei RAS1* is essential, and the analysis of Ras/Cdc42/Rac pathways has therefore depended on the use of constitutively active or inactive mutant *RAS* alleles.

Finally, in the basidiomycete model organism *U. maydis*, which encodes duplicate Ras proteins as well as single Cdc42 and Rac homologs, *Um*Ras proteins have been shown to act through cAMP/PKA and MAPK cascades and to be involved in filamentation and pheromone sensing [25]. *Um*Rac1 is required for filamentation and the yeast-hyphal transition, while *Um*Cdc42 plays a dominant role in organizing the proteins involved in yeast phase cytokinesis [56]. While *Um*Rac1 and *Um*Cdc42 play overlapping roles, with their loss being synthetically lethal, the connections between Ras function and Rac and Cdc42-mediated morphogenesis remain undefined [56].

**Figure 7:**
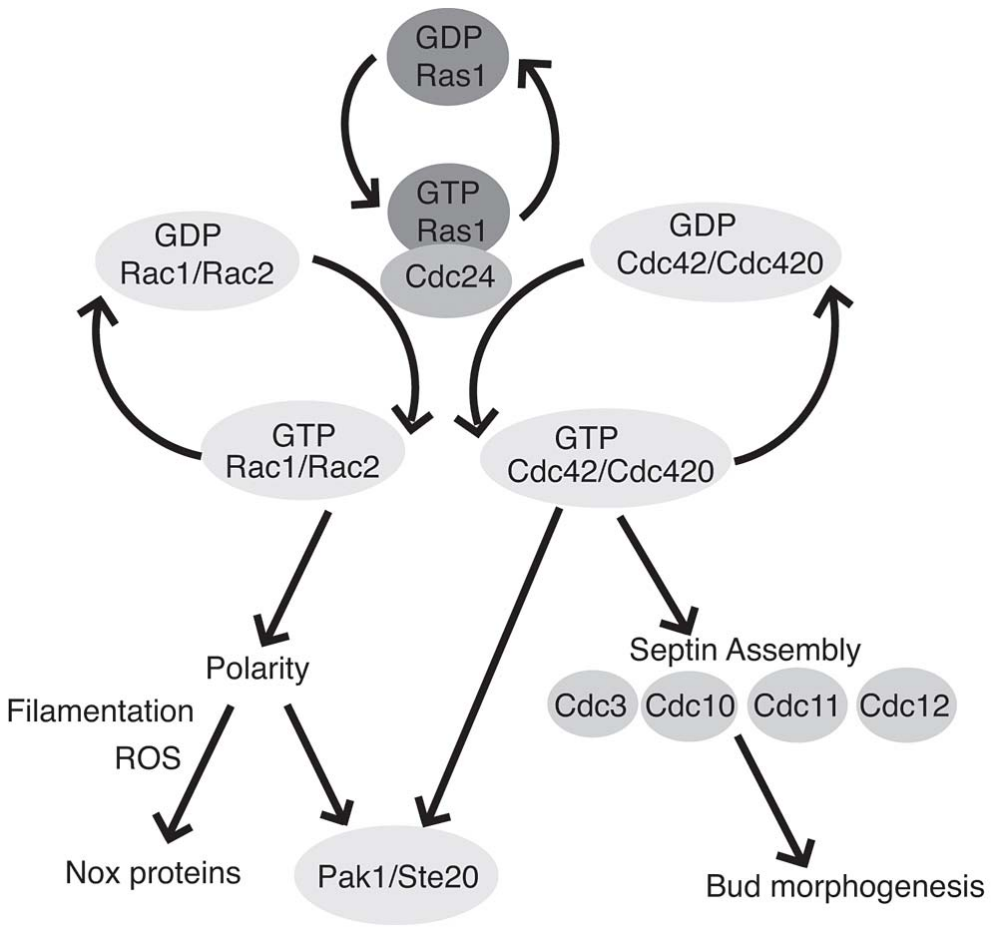
Ras1 acts through Cdc42 and Rac paralogs to regulate polarized growth and morphogenesis. GTP-bound Ras1 preferentially interacts with the Rho-GEF Cdc24 and mediates the activation of Rac1/Rac2 and Cdc42/Cdc420. The Rac paralogs primarily regulate polarized growth and organize the localization of ROS during filamentous growth, possibly through interaction with as yet unidentified Nox proteins. Cdc42 paralogs are primarily involved in cytokinesis and bud morphogenesis via the organization of the septin proteins. Additionally, Rac and Cdc42 paralogs physically interact with both Pak1 and Ste20.

Therefore, although it is strongly suggested by the literature that fungal Ras proteins can act through both Rac and Cdc42 to mediate independent morphogenetic events, it had not yet been definitively demonstrated. We took advantage of the plasticity of the *C. neoformans* requirement for these proteins to examine this interaction in the context of a *ras1Δ* mutant background. Based on this analysis we have further refined the model of the Ras1 signal transduction pathway in *C. neoformans* (Figure 7).

Although the loss of either Ras1 or Cdc24 function in *C. neoformans* impairs growth at high temperature, both *ras1Δ* and *cdc24Δ* mutants proliferate at rates comparable to wild type cells in the absence of stress [17]. We have previously demonstrated that *CDC42* paralogs are not essential for *C. neoformans* proliferation, but *cdc42Δ cdc420Δ* double mutant cells exhibit significant morphological defects, even at 25°C [19]. Additionally, Cdc42 activity is dose dependent: the induction of *CDC42* expression is required to facilitate growth at 37°C [19]. Finally, we demonstrate here that the loss of *RAS1* impairs, but does not completely ablate, GTP binding by Cdc42. Together, these data suggest that basal levels of Cdc42 activity persist even in the absence of Ras1 or Cdc24 function, and that this basal activity is sufficient to support growth in the absence of stress. Moreover, because Rac function is likewise impaired by the loss of Ras, we predict that basal levels of Rac activity also help to support the growth of the *ras1Δ* and *cdc24Δ* mutants in the absence of stress.

An emerging theme among filamentous fungi is the functional overlap of Rac and Cdc42 homologs. The two have been shown to play overlapping roles in polarized growth and septation, and the loss of both GTPases is often synthetically lethal [18], [56], [57]. However, mutant analysis in *C. albicans* has demonstrated the potential for specialization of function between the two classes of GTPases [20]. Likewise, our data are consistent with Rac and Cdc42 protein functional specialization in *C. neoformans*. While *CDC42* paralog but not *RAC* paralog over-expression was sufficient to restore bud morphogenesis, *RAC* paralog but not *CDC42* paralog over-expression was sufficient to restore *ras1Δ* yeast phase ploidy and yeast- and hyphal- phase polarized growth.

Previously, we demonstrated significant overlap in Rho-GTPase effector interactions. For example, both Rac1 and Cdc420 have been shown to interact with the p21-activated kinase Ste20 [22], [58]. Similar overlap has been suggested in *C. albicans*, in which both Cdc42 and Rac1 have been suggested to interact with the PAK Cst20 [50], [55]. Significantly, we have previously demonstrated that *STE20* is dispensable for septin organization, suggesting that Cdc42 paralogs may mediate septin-independent processes via Ste20 [19]. Future work will therefore be required to examine the specific roles of Rac and Cdc42 paralogs in the activation of Ste20 and other downstream effectors.

Based on our hypothesized roles for Ras1 in regulating Cdc42 activity and, indirectly, septin activity, we predicted that *ras1Δ* cells might accumulate defects in septin-mediated processes, including, bud morphology, cytokinesis, and thermotolerance. We observed that the localization of a Cdc11-fusion protein was indeed altered in the absence of *RAS1* at 37°, but not 30°C, consistent with impaired Cdc42-mediated septin assembly. The temperature-dependence of Cdc11 localization in the *ras1Δ* background is consistent with the temperature sensitivity of *ras1Δ, cdc24Δ, cdc42Δ* and septin-deficient mutants.

 In addition to altered septin function, cells deficient for *RAS1* exhibit gross defects in polarized growth. Mutant *ras1Δ* cells are larger than wild type at 30°C, are defective in the repolarization of actin following exposure to high temperature (37°C) or the actin-destabilizing agent Latrunculin B, and fail to grow in a polarized fashion under mating conditions [17], [19]. This role for Ras in polarized growth is conserved among filamentous fungi, as well as in mammalian cells. Here we demonstrate that the Rac proteins mediate the effects of Ras in the polarized growth of *C. neoformans* cells. Moreover, the cell polarity events mediated by the Ras/Rac signaling axis are likely due to alterations in the subcellular localization of reactive oxygen species (ROS) [23]. This may occur either through the directed localization of NADPH oxidase (Nox) proteins, as has been reported in *A. nidulans*, or through compartmentalized regulation of Nox activity, as demonstrated in *E. festucae* and *C. purpurea* [59]–[61]. The effects of *C. neoformans* Ras and Rac proteins on fungal cell polarity directly influence hyphal development and yeast cell size.

After 4 hours at 37°C, we observed defects in bud morphology in the *ras1Δ* mutant, including the apparent failure of cytokinesis prior to the emergence of the next bud. Bud emergence and cell cycle control are tightly linked in *S. cerevisiae* via the phosphorylation of cyclins [38], [62]. Moreover, it has recently been demonstrated that the septin proteins play a key role in the regulation of this process by stabilizing the kinase Hsl1 at the bud neck [63]. Consistent with this role for bud neck proteins in the regulation of cell cycle events, we found that *ras1Δ* mutant cells have altered ploidy. However, the over-expression of *CDC42* paralogs, which stabilize the organization of septin proteins in the *ras1Δ* mutant, had no effect on *ras1Δ* ploidy. Instead, the over-expression of *RAC2*, which restored yeast phase cell size but not morphology, restored *ras1Δ* ploidy. This suggests that *ras1Δ* defects in cell cycle control occur independently of bud morphogenesis defects. Additionally, it suggests that *ras1Δ* yeast phase defects in cell size may be the result of defects in both polarized growth (as observed by their increased sensitivity to actin polymerization inhibitors) and in ploidy (as diploid cells are larger than haploid cells). Future work will explore the role of Ras and Rac proteins in *C. neoformans* cell cycle control. This observation is likely very important in the pathogenesis of *C. neoformans* infections, given recent data associating alterations in ploidy with changes in virulence and antifungal drug resistance [64], [65]. Additionally, polyploid titan cells are a newly described morphological variant of *C. neoformans* that may favor persistence and dormancy in lung tissue [66], [67].

By encoding duplicate paralogs of Ras, Rac, and Cdc42 proteins, *C. neoformans* provides a unique system in which to examine interactions among these signaling proteins *in vivo*. We have taken advantage of this system to define the relative contributions of the Rac and Cdc42 proteins to Ras-mediated morphogenesis and thermotolerance. These microbial phenotypes, and therefore the Ras/Cdc42/Rac signaling axes, are essential components of pathogen survival in the host and the subsequent establishment of human disease.

## Materials and Methods

### Strains, media, and growth conditions

*C. neoformans* strains used in this study are listed in Table 2. Strains were incubated on YPD medium [68], V8 mating medium [69], or MS mating medium [70]. ERB011 was obtained by mating ERB005 with ERB007 and dissection of the resulting spores. ERB018, ERB023, and ERB024 were generated by mating LK001 with ERB002, ERB010, or CSB40, respectively, and spore dissection. Strain ERB039 was generated by biolistic transformation with the plasmid pLKB95. Strain ERB122 was generated by mating between ERB039 and KN99a and dissection of the resulting spores [71]. All other strains were generated via biolistic transformation according to the protocol developed by Toffaletti *et al.* [72]. Mating assays were performed by co-culturing strains of opposite mating type on MS mating medium in the dark at 25°C for 4 days. For morphogenesis experiments, cells were inoculated into liquid YPD (2% glucose) and grown to mid-log phase at 30°C, shaking at 150 RPM. Cultures were split and refreshed with media pre-warmed to 30 or 37°C, then incubated at the indicated temperature at 150 RPM for four hours or over night, as indicated. For galactose inducing conditions, 3% galactose was added to YP broth, and the medium was refreshed every 8–12 hours to maintain inducing galactose levels. Mating assays were performed by co-culturing strains of opposite mating type on V8 or MS mating medium in the dark at 25°C.

**Table 2:** Strains in this work.

Strain	Genotype	Source
H99	*MATα*	[80]
KN99a	*MAT* ***a***	[71]
LCC1	*MATα ras1::ade2*	[2]
CBN45	*MATα ras1::neo*	[12]
MWC14	*MATα ras1::ade2 ras2::ura5*	[21]
KN4B7#16	***a*** *AA* ***a***	[39]
ERB057	*MATα ras1::ade2+pHCDC42-NEO*	This study
ERB058	*MATα ras1::ade2+pHCDC420-NEO*	This study
ERB124	*MATα ras1::ade2+pHRAC1-NAT*	This study
ERB067	*MATα ras1::neo+pHRAC2-NAT*	This study
ERB039	*MATα pHCdc11-mCherry-HYG*	This study
ERB122	*MAT* ***a*** * pHCDC11-mCherry-HYG*	This study
ERB052	*MATα ras1::ade2+pHCDC11-mCherry-HYG*	This study
ERB126	*MATα ras1::ade2+pHCDC42-NEO+pHCDC11-mCherry-HYG*	This study
ERB128	*MATα ras1::ade2+pHCDC420-neo+pHCDC11-mCherry-HYG*	This study
ERB071	*MATα ras1::ade2+pHRAC2-NAT+pHCDC11-mCherry-HYG*	This study
CNB300	*MATα ras1::ade2+pG7RAS1-NAT*	This study

### Molecular biology

To generate *cdc42Δ* and *cdc420Δ* deletion mutants using dominant selectable markers, PCR overlap extension was used to replace the entire *CDC42* and *CDC420* open reading frames with the neomycin or nourseothricin resistance cassettes in the H99α or KN99**a** strains as described [73], [74]. Genomic integration was performed using the biolistic transformation method as described [72], [75]. Deletion strains were confirmed by PCR, Southern blot analysis, and RT-PCR. Primers used for PCR overlap extension include the following (5′ to 3′, sequence for *NAT* gene underlined): *CDC42* Left flank: AA1468: GCCAGGGGTTGCACCGGGA and AA1409: GTCATAGCTGTTTCCTGTGTCTGCATGGTTGGCTAGG; Right flank AA1412: CTGGCCGTCGTTTTACGTGCCTCATCCTTTGAAGAC and AA1469: TCGCTGTACATCGTCGAATC; sequence for *NAT* gene for *cdc42::NAT* allele: AA1410: CCTAGCCAACCATGCAGACACAGGAAACAGCTATGAC and AA1411: GTCTTCAAAGGATGAGGCACGTAAAACGACGGCCAG ; *CDC420* Left flank: AA1466: AGAGAGGGGGAGTGGAGGTA; and AA1405: GTCATAGCTGTTTCCTGTATCTCTTAAAGTTGCGGGG; Right flank AA1408: CTGGCCGTCGTTTTACGCTTGATCCTCTAGTACAC and AA1467: TGCAATCCTCGAACACTACG; *NAT* gene for *cdc420::NAT* allele: AA1406: CCCCGCAACTTTAAGAGATACAGGAAACAGCTATGAC and AA1407: GTGTACTAGAGGATCAAGCGTAAAACGACGGCCAG .

To generate the *rac1Δ* strain, the following primers with regions homologous to flanks surrounding the hygromycin marker in pHyg-KB2 were used: AA1354: CGCCAACACTTGCTGCCGCTC; AA1814: GTCATAGCTGTTTCCTGTCTGGGCTGTTGCGCTATGCC; AA1815: GGCATAGCGCAACAGCCCAGACAGGAAACAGCTATGAC; AA1816: CCTCATTTGGCATTGCTCAGCGTAAAACGACGGCCAG; AA1817: CTGGCCGTCGTTTTACGCTGAGCAATGCCAAATGAGG; AA1359: GCAGGCGAAGAGCGGATGG. To generate the *rac2Δ* strains, the following primers with regions homologous to the *m13* flanks surrounding the *NEO* and NAT markers were used: AA3122: GTTGGGCCACCATCATTACT; AA3194: TACCATCATCCTCTCCTCCGTTGCTCTCGTTGAGGATTGA; AA319: TCAATCCTCAACGAGAGCAACGGAGGAGAGGATGATGGTA; AA3196: AGCTGTACCCTTGTCCGCTACGACAGCATCGCCAGTCACTA; AA3197: TAGTGACTGGCGATGCTGTCGTAGCGGACAAGGGTACAGCT; AA3127:CTCCTTCCACCCACCACTTA.

To generate over-expression constructs, the open reading frames of *C. neoformans* Serotype A *CDC42*, *CDC420*, *RAC1*, and *RAC2* were amplified by PCR using primers that incorporated the appropriate restriction sites in frame at the 5′ and 3′ ends. These primers were: For *CDC42*: AA1517: CGGGATCCCATGCAGACAATCAAGTGTG; AA1519: CGGGATCCCGAGAAGGGGGAGTCTGGAAC; For *CDC420*: AA1637: CGCGGATCCATGCAGACTATCAAATGTG; AA1518: CGGGATCCCCAAAGTGGTCGTTGGAGGAT; For *RAC1*: AA1959: GCCGGTACCCGCCAACACTTGCTGCCGCTC; AA1960: GGCGAGCTCCTGGGCTGTTGCGCTATGCCG; For *RAC2*:AA1928: GGGCCCAGATCTATGGCCATGCAGAGTATC; AA3090: CCCGGGAGATCTGATCCGTGCTTGGTTTTTGT.

Over-expression constructs were then generated by TA cloning and/or BamH1 ligation into the plasmid pCN19, containing the *HIS3* promoter sequence, the actin terminator sequence, and the nourseothricin selection marker, to generate the plasmids pERB01, pERB02, pJN03, and pERB04, respectively [76]. The inducible *RAS1* expression construct was generated by cloning the *RAS1* open reading frame into the plasmid pCN68, containing the *GAL4* promoter sequence, the actin terminator sequence, and the nourseothricin selection marker. *C. neoformans* strains over-expressing *CDC42*, *CDC420*, *RAC1*, or *RAC2* were generated in *RAS1* and *ras1::ade2* (strain LCC1, [2]) backgrounds by biolistic transformation with the following plasmids: pERB01, pERB02, pJN03, or pERB04. Plasmid LKB95 expressing the Cdc11-mCherry fusion protein from the *GPD* promoter was cloned as follows. First, the open reading frame for *CDC11*+terminator (337 bp from STOP) was PCR-amplified from the genomic DNA of H99 with flanking restriction enzyme sequences for NheI and PacI. The primers for the PCR were JOHE24124 and JOHE24125. The PCR product was cleaved with NheI and PacI and ligated into plasmid LKB55 (a derivative of LKB49 with *NEO* replaced by *HYG*), which had been cleaved with NheI/PacI and CIP-treated [77]. Genomic integration was performed using the biolistic transformation method as previously described [72], [75].

### Microscopy

Differential interference microscopy (DIC) and fluorescent images were captured with a Zeiss Axio Imager A1 fluorescent microscope equipped with an AxioCam MRM digital camera. Cells were fixed with 9% microfiltered formaldehyde for 10 minutes, washed three times with 1XPBS, permeabilized with 1% Triton-PBS for 10 minutes, and washed three times with 1XPBS. Cells were stained with Calcofluor or Rhodamine-conjugated Phalloidin. For cells containing the mCherry construct, localization was examined in live cells. Cell counts were performed using ImageJ software. For cells incubated at 37°C for 4 hours, more than 100 cells with buds were counted for the analysis of bud morphology. Reported n values represent the total number of budded and unbudded cells observed. In order to image mating structures, agar plugs were excised from mating reactions and thinly sliced in preparation for slide squash. The plugs were fixed with 9% microfiltered formaldehyde for 30 min, washed three times with 1XPBS, permeabilized for 30 min in 1% Triton-PBS, and washed three times with 1XPBS. Filaments were stained with Dapi or Calcofluor and visualized by slide squash. For the visualization of clamp cell morphology, hypha were fixed and permeabilized as described and then stained with calcofluor to facilitate visualization of the entire clamp structure. Hyphal clamp cell morphology was observed for more than 150 clamp cells in each unilateral cross (*RAS1α×RAS1* ***a***, *ras1Δ+RAC1xRAS1* ***a***, and *ras1Δ+RAC2xRAS1* ***a***) according to the following conditions: 1) In order to account for hyphal context, only structures on hyphae in which three or more structures were fully visible were counted; 2) Structures were considered fully visible when they were parallel to the plain of the slide. Serial live images of *C. neoformans* mating events were observed on MS medium using cells that had been allowed to grow to log-phase overnight and then co-cultured overnight at a low density (<10^6^ cells/mL) on MS agar patch slides. Images were acquired using at 63× magnification every 15 minutes.

### RNA extraction, cDNA preparation, and RT-PCR

Expression levels for each of the over-expression constructs were determined by RT-PCR. Mid-log phase cells incubated at 30°C in YPD were collected by centrifugation and flash frozen on dry ice. Total RNA was extracted from lyophilized cells using the Qiagen RNA extraction kit and the ‘Purification of total RNA from plant cells, tissue, and filamentous fungi’ protocol (2006). cDNA was prepared using the Clontech Advantage RT-for-PCR kit (2006). Primer specificity was verified by qPCR in wild-type and deletion strains. RT-PCR was performed as described, with annealing at 50°C [78]. Primers used were as follows: *CDC42*: Forward: AA778: CGTCCCCGCACTTATTGTC; Reverse: AA782: AGTCGCCATAGGGGGTTCTAAT. *CDC420* Forward: AA1768: TTTGAGGGATGATCCAAAGCA; Reverse: AA1769: CATTCTTCAACCCCTTTTGC. *RAC1* Forward: AA1853: CCGAACCAAATGGTATCCTG; Reverse: AA1854: TTAGGGTTGAGGACTGTCCG. *RAC2* Forward: AA1851: TGTCAAAACTTGGATCCCCG; Reverse: AA3021: CAAGCCTTTTTGCGTCCGACTAGAAG. *GPD* Forward: AA301: AGTATGACTCCAACAATGGTCG; Reverse: AA302: AGACAAACATCGGAGCATCAGC.

Expression levels were calculated using the ΔΔCt method, as described [79].

### Pak activation assay

The *in vitro* activity of *C. neoformans* Cdc42 and Rac2 proteins was determined using a commercially available Cdc42 Pull-Down and Activation assay (Pierce Thermo Scientific). Wild type and *ras1Δ* mutant strains expressing *GFP-CDC42* and *GFP-RAC2* alleles (*CBN302*, *CBN337*, *ERB130*, and *ERB74*) were grown overnight in YPD medium. Strains were diluted five-fold into pre-warmed YPD media (30°C or 37°C) and grown for four hours. For each culture, 20 O.D._600_ equivalents were centrifuged and resuspended in 0.5 ml Lysis/Binding/Wash Buffer (LBW; 25 mM Tris-HCl, pH 7.2, 150 mM NaCl, 5 mM MgCl_2_, 1% NP-40 and 5% glycerol) containing 2× protease inhibitors (Complete Mini, EDTA-free, Roche) and phosphatase inhibitors (Phos-STOP, Roche). To lyse the cells, the supernatant was removed, and cell pellet was disrupted using 0.5 ml of glass beads in a Mini-Beadbeater-16 (Biospec) 6 times for 30 sec each with 1 min incubations on ice in between. The lysate was extracted 3 times with 0.5 ml of LBW, incubated for 30 min at 4°C to enhance solubilization, and centrifuged for 10 min. Duplicate cell lysates were combined, and the protein concentration was determined using Precision Red (Cytoskeleton). Samples were aliquoted for loading controls and for the Cdc42 activation assay. The Cdc42 activation assay was performed according to the instructions provided by the manufacturer (Pierce Thermo Scientific). Each experimental sample lysate was incubated with GST-PAK-PBD to specifically bind the GTP-bound, active Cdc42/Rac2 and precipitated with glutathione beads. Following the assay, bound proteins were eluted from the glutathione-agarose beads by the addition of 50 µl 2× Laemmli sample buffer. Experimental and control samples were separated by NuPAGE electrophoresis, transferred to PVDF, and immunoblotted with anti-GFP antibodies. Signal intensity ratios were determined using imageJ.

### Western blot analysis

Samples were heated to 95°C for 4 min and 10 or 25 µl (control lysate and assay sample, respectively) was loaded and separated on a NuPAGE 4–12% Bis-Tris gel (Invitrogen) using MES running buffer. Samples were electrophoretically transferred to Invitrolon PVDF membrane (Invitrogen). The membrane blot was incubated with Starting Block T20 (Pierce) for 1 hr, incubated with anti-GFP antibody (1/4000 dilution, Pierce Thermo Scientific) for 1 hr, washed 5×5 min with TBST, incubated with an anti-mouse peroxidase-conjugated secondary antibody (1/50,000 dilution, Jackson Labs), and washed 5×5 min with TBST. Reactive proteins were detected by ECL Prime Western Blotting Reagents (GE).

## Supporting Information

Figure S1 Haploid filamentation is induced in *C. neoformans* by the dominant active Ras1^Q67L^ protein. The over-expression of a dominant active allele of *RAS1*, *RAS1^Q67L^*, results in a gain of function. *RAS1^Q67L^* strains filament on FA, while wild type strains do not. This gain of function is reduced but not eliminated by the loss of *RAC* paralogs. The over-expression of either *RAC* paralogs or *CDC42* paralogs is insufficient to induce filamentous growth.
(TIF)

Figure S2 Septin proteins localize to the site of peg/clamp fusion. The Cdc10-mCherry fusion construct localizes to sites of peg/clamp fusion during filamentous growth. Cdc10-mcherry mating type α cells were co-incubated with wild type mating type **a** cells on MS mating medium for 7 days. Plugs were fixed, counter-stained with calcofluor white, and prepared for imaging as discussed in the Materials and Methods.
(TIF)
