# Supplementary figures and images for: Ras1 Acts through Duplicated Cdc42 and Rac Proteins to Regulate Morphogenesis and Pathogenesis in the Human Fungal Pathogen Cryptococcus neoformans
Source: PLoS Genet. 2013 Aug 8;9(8):e1003687. doi: 10.1371/journal.pgen.1003687 (PMC3738472; doi:10.1371/journal.pgen.1003687)

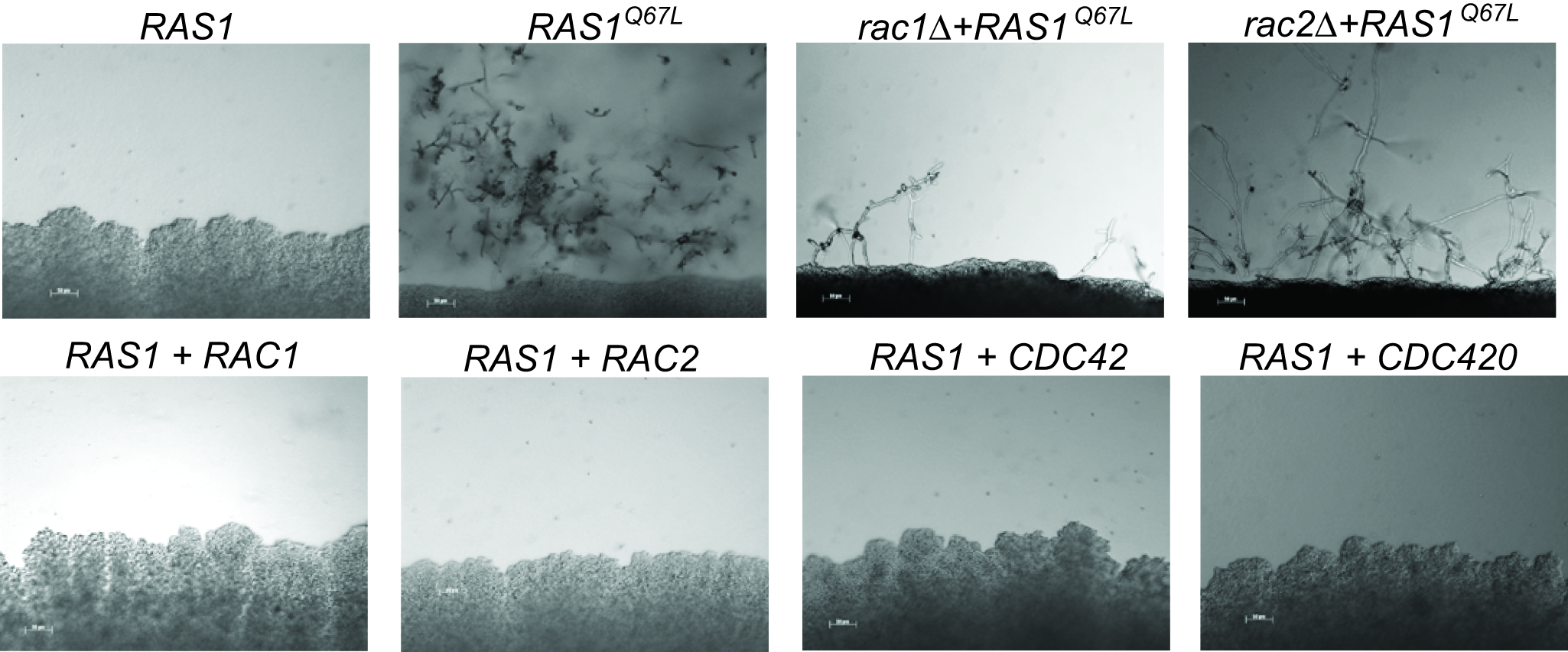

Supplement: Figure S1 — Haploid filamentation is induced in C. neoformans by the dominant active Ras1Q67L protein. The over-expression of a dominant active allele of RAS1, RAS1Q67L, results in a gain of function. RAS1Q67L strains filament on FA, while wild type strains do not. This gain of function is reduced but not eliminated by the loss of RAC paralogs. The over-expression of either RAC paralogs or CDC42 paralogs is insufficient to induce filamentous growth. (TIF) [file pgen.1003687.s001.tif]

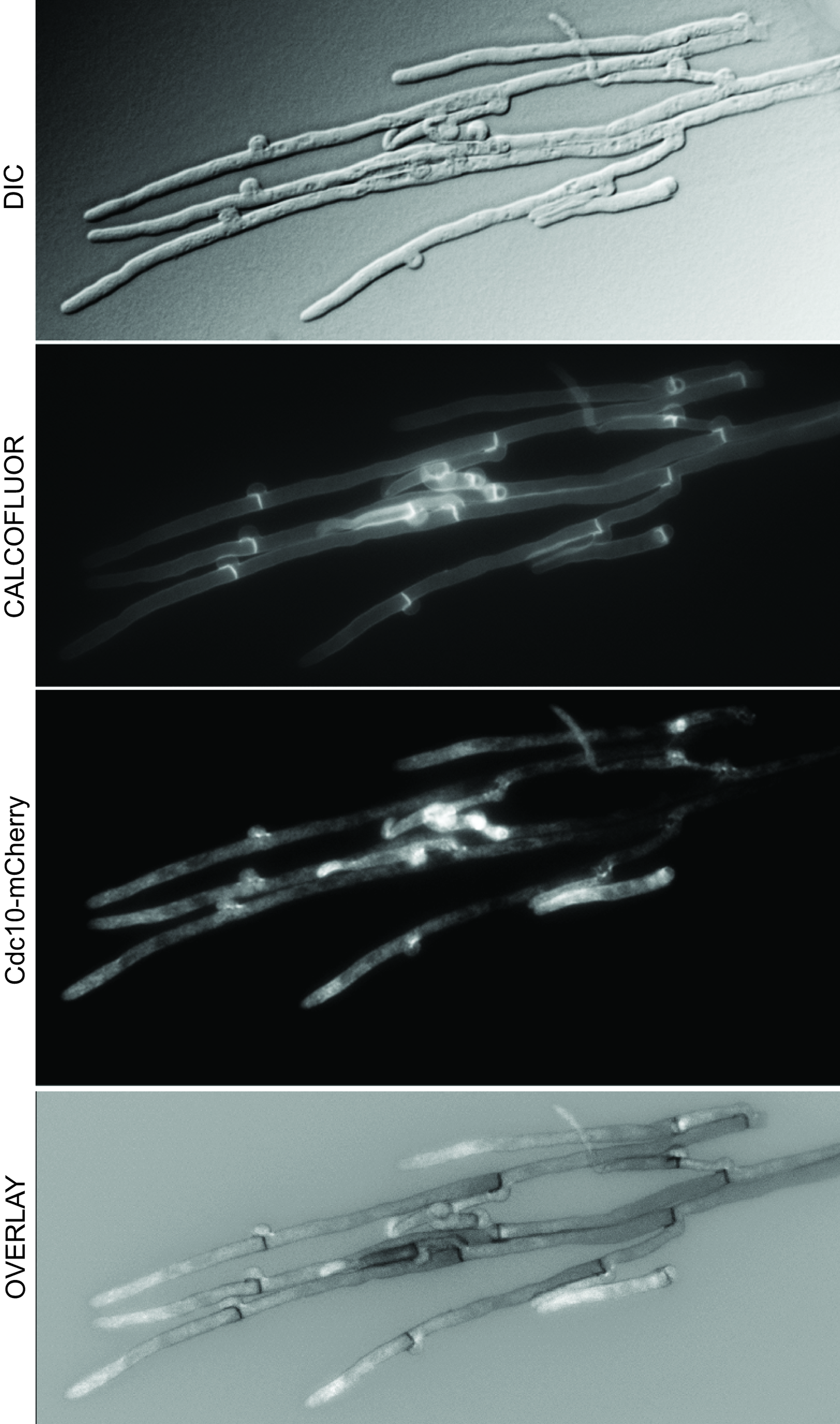

Supplement: Figure S2 — Septin proteins localize to the site of peg/clamp fusion. The Cdc10-mCherry fusion construct localizes to sites of peg/clamp fusion during filamentous growth. Cdc10-mcherry mating type α cells were co-incubated with wild type mating type a cells on MS mating medium for 7 days. Plugs were fixed, counter-stained with calcofluor white, and prepared for imaging as discussed in the Materials and Methods. (TIF) [file pgen.1003687.s002.tif]
